# Cyclodextrins and Their Polymers Affect Human Serum Albumin’s Interaction with Drugs Used in the Treatment of Pulmonary Infections

**DOI:** 10.3390/pharmaceutics15061598

**Published:** 2023-05-25

**Authors:** Anna A. Skuredina, Linara R. Yakupova, Tatiana Yu. Kopnova, Irina M. Le-Deygen, Natalya G. Belogurova, Elena V. Kudryashova

**Affiliations:** Department of Chemistry, Lomonosov Moscow State University, 119991 Moscow, Russia; skuredinanna@gmail.com (A.A.S.);

**Keywords:** cyclodextrins, antibacterial agents, pulmonary infections, human serum albumin

## Abstract

Respiratory infectious diseases have challenged medical communities and researchers. Ceftriaxone, meropenem and levofloxacin are widely used for bacterial infection treatment, although they possess severe side effects. To overcome this, we propose cyclodextrin (CD) and CD-based polymers as a drug delivery system for the drugs under consideration. CD polymers demonstrate higher binding affinity for levofloxacin (Ka ≈ 10^5^ M) compared to drug–CD complexes. CDs slightly alter the drugs’ affinity for human serum albumin (HSA), whereas CD polymers increase the drugs’ binding affinity up to 100 times. The most significant effect was observed for more the hydrophilic drugs ceftriaxone and meropenem. The drug’s encapsulation in CD carriers leads to a decrease in the degree of change in the protein’s secondary structure. The drug–CD carrier–HSA complexes demonstrate satisfying antibacterial activity in vitro, and even a high binding affinity does not decrease the drug’s microbiological properties after 24 h. The proposed carriers are promising for a drug form with a prolonged drug release.

## 1. Introduction

The COVID-19 pandemic continues to challenge medical staff and have worldwide impacts on healthcare organizations. Viral infection weakens the immune system, and patients become more susceptible to bacterial infections [1]. Thus, approximately 15–30% of hospitalized patients suffering from SARS-CoV-2 infection develop further respiratory issues [2]. Moreover, COVID-19-associated pneumonia causes long-term severe respiratory disorders [3] and chronic illnesses even after one year of treatment [4].

Antimicrobials are usually prescribed to prevent secondary infection or to treat co-infections in hospitals in an empirical setting. These drugs include broad-spectrum antibiotics and antibacterial agents active against *Streptococcus pneumoniae*, *Staphylococcus aureus*, etc. [5,6]. However, the International Coordination Group on Antimicrobial Resistance calls for rational drug consumption due to the rising global antimicrobial resistance crisis [7]. Moreover, long-term treatment with antibacterial medication leads to the development of severe side effects, including gastrointestinal disorder, photosensitivity, severe headache, etc. [8]. Hence, new drug forms with high efficiencies at lower doses are urgently required. As a very promising approach, one could consider drug encapsulation for specifically designed drug delivery systems.

Among all the drug delivery systems, cyclodextrins (CDs) are one of the most prospective choices due to their ability to form non-covalent guest–host complexes that improve drug solubility, stability and bioactivity [9]. CDs are torus-shaped oligosaccharides consisting of 6–8 D-glucopyranose units, where –OH groups can be modified to obtain the desired properties [10]. Drug–CD derivative complexes are well-studied in the literature [11,12]. Nevertheless, CD’s influence on drug properties is limited, and newly emerged, synthesized CD-based polymers [13,14] appear to be highly efficient thanks to the combination of the advantages of both CD and polymers: a CD cavity to form a guest–host complex and a polymeric network that can additionally maintain the drug molecule [15,16]. These studies mainly focus on the CD polymers’ physical–chemical properties and do not consider the carrier’s ability to influence the drug’s interaction with biological macromolecules. However, this research is crucial to the understanding of how the carrier might change the drug’s properties in vivo.

For instance, the intravenous administration of drug forms used in antibiotic treatment induces the interaction of the active molecule with blood components. Human serum albumin (HSA) is the main blood protein that is responsible for the transport of numerous endogenous compounds such as amino acids, hormones, fatty acids, antimicrobial agents, etc. [17,18]. A change in the binding force and/or binding affinity between the drug and HSA might alter the main pharmacokinetic parameters, such as the volume of drug distribution, clearance, and/or elimination, which consequently might significantly affect the therapeutic effect. Thus, the understanding how CD-based polymers change drug–albumin binding is crucial to the development of novel highly efficient drug forms.

Here, we studied the three-component system (drug–CD carrier–HSA) to uncover the influence of CD and CD-based polymers on the interaction of drug molecules with HSA: the binding affinity along with the structural changes in albumin. Since cephalosporins, β-lactams and fluoroquinolones are three groups that are widely used to treat COVID-associated respiratory bacterial infection [19,20], we chose the antibacterial molecule (AM) from each class. Furthermore, we discuss the in vitro properties of the three-component system (drug–CD carrier–HSA) to understand if it has potential to be used against bacterial infection. We hope our research will be useful for understanding the potential of CD-based carriers as drug delivery systems for intravenous administration. 

## 2. Materials and Methods

### 2.1. Materials

The antibacterial molecules ceftriaxone (Cef), meropenem (Mer), levofloxacin (Lev), 2-hydroxypropyl-β-cyclodextrin (HPCD; the degree of substitution per one D-glucopyranose unit is 0.5–1.3), methyl-β-cyclodextrin (MCD; the degree of substitution per one D-glucopyranose unit is 1.5–2.1), 1,6-hexamethylene di-isocyanate (HMD), dimethyl sulfoxide, citric acid, succinic anhydride, human serum albumin and NaH_2_PO_4_ were all from Sigma-Aldrich (St. Louis, MO, USA). Tablets were used for the preparation of sodium phosphate buffer solution with pH 7.4 (Pan-Eco, Moscow, Russia).

*Escherichia coli* ATCC 25,922 was from the Russian National Collection of Industrial Microorganisms (VKPM), Scientific Center «Kurchatov Institute» (Moscow, Russia).

### 2.2. Synthesis of CD-Based Polymers

HPCD-based polymers linked by citric acid and succinic anhydride were obtained according to [21]. Briefly, the required amount of HPCD solution (C = 60 mg/mL) was mixed with a catalyst (NaH_2_PO_4_) and heated to 140 °C. Next, a certain quantity of aqueous linker solution (3M citric acid or succinic anhydride) was inserted dropwise into the reaction mixture, which was then stirred for 1.5 h. The molar HPCD:linker ratios were 1:15 for both linkers.

The synthesis of HPCD and MCD polymers linked by 1,6-hexamethylene di-isocyanate (HMD) were implemented following [15]. Shortly, the required quantity of DMSO was added dropwise into the warm, aqueous CD solution (C = 100 mg/mL) to compose a solvent ratio of 1:1 by volume. After 5 min, the linker was added to the reaction mixture with intensive stirring. The CD:linker molar ratio was 1:5 and 1:3 for HPCD and MCD, correspondingly.

The purification of all CD polymers was performed by dialysis (cut-off 12-kDa-molecular-weight membrane, Serva, Heidelberg, Germany) at room temperature. The samples were dried for 24 h at 25 °C in Petri dishes using a thermostat (ES-20, Biosan, Riga, Latvia).

The CD torus concentration in CD polymers was determined by FTIR spectroscopy using the calibration dependence for the 1032 cm^−1^ band (C–O–C bond in CD torus).

### 2.3. Antibacterial Molecule–CD Carrier Complex Formation

AM–CD or AM–CD polymer complexes were obtained by mixing aqueous solutions of individual components in different molar ratios (AM:CD torus = from 0.1 to 10) at pH 4.0 (HCl) for levofloxacin and at pH 7.4 for ceftriaxone and meropenem (sodium phosphate buffer solution). The samples were stored at 37 °C for 30 min. For the experiments involving protein (HSA), the complexes’ solutions were diluted 100 times with a sodium phosphate buffer solution (pH 7.4).

The calculation of the AM–CD polymer binding constants (Ka) considering the equilibrium AM + nCD ↔ AM × nCD (n is the number of CD torus polymers per drug molecule) was conducted by Equation (1) [22,23]:

(1) $$K_{a}=\frac{\left[\mathrm{AM}\cdot \mathrm{nCD}\right]}{{\left[\mathrm{CD}\right]}^{n}\left[\mathrm{AM}\right]}$$

We varied the concentration of CD tori in the CD polymers and analyzed the changes in the intensity of the AM emission spectra by Hill’s linearization in the n-binding site model (2) [24]:

(2) $$\log\frac{\theta }{1-\theta }=n\cdot \log\left[\mathrm{CD}\right]+{\mathrm{logK}}_{a}$$

where θ is a fraction of the bounded AM molecules calculated as (3)

(3) $$\theta =\frac{\xi -\xi_{o}}{\xi_{\infty }-\xi_{o}}$$

where $\xi_{0}$ and ξ are the peak intensities of the fluorescence spectra of Lev in the absence and the presence of CD polymer, and $\xi_{\infty }$ is the intensity of the horizontal asymptote.

### 2.4. Preparation of Two-Component (AM–HSA) and Three-Component (AM–CD Carrier–HSA) Systems

After mixing the AM and HSA solutions (sodium phosphate buffer solution, pH 7.4), the sample volume was adjusted to 1 mL. The HSA concentration was maintained in all samples (0.06 mM), while the molar excess of the AM was varied in the range of 0.25–20. The complexes were incubated at 37 °C with constant stirring for 1 h. Directly before the measurement of fluorescent spectra, the solutions were diluted 3 times with sodium phosphate buffer solution (pH 7.4) to obtain the final HSA concentration of 0.02 mM.

### 2.5. FTIR Spectroscopy

The FTIR spectra were recorded using a Tensor 27 spectrometer (Bruker, Ettlingen, Germany) equipped with an attenuated total reflection cell (Bruker, Ettlingen, Germany), ZnSe crystal, an MCT detector cooled with liquid N_2_ and dry air by the air compressor (Jun-Air, Munich, Germany), and a thermostat (Huber, Offenburg, Germany). Each FTIR spectrum was registered 3 times (40 μL of the sample; 210 scans in total) in the range of 2200–850 cm^−1^ (1 cm^−1^ resolution) at 22 °C. The data were analyzed by the Opus 7.0 software (Bruker, Ettlingen, Germany). 

The secondary structure of the HSA and HSA–AM complexes was determined by spectrum analysis in the range 1700–1600 cm^−1^. The second derivative of the spectrum was taken to define the main bands. The deconvolution of spectra was performed with the Levenberg–Marquardt algorithm. The residual RMS error was less than 0.00001.

### 2.6. Fluorescence Spectroscopy

Fluorescence measurements were conducted on a Varian Cary Eclipse spectrophotometer (Agilent Technologies, Santa Clara, CA, USA). The emission spectra of HSA were registered at 37 ± 0.1 °C in a 1 cm-thick cuvette at the excitation wavelength of 280 nm in the range of 290−555 nm with a step of 1 nm. The protein concentration in all samples was maintained at 0.02 mM (solution phosphate buffer, pH = 7.4). The emission spectra of Lev were registered at 37 ± 0.1 °C in a 1 cm-thick cuvette at the excitation wavelength of 345 nm in the range of 360−600 nm with a step of 0.5 nm.

### 2.7. Nanoparticle Tracking Analysis (NTA)

NTA was performed by the Nanosight LM10-HS device (Nanosight Ltd., Malvern, UK). The CD-based polymers were dissolved in Milli-Q purified water (1–2 mg/mL), then were diluted to obtain the solutions with ca. 10^8^ particles/mL. The measurements were performed 5 times for each sample. The values are provided with standard deviations.

CD polymer’s average molecular weight (Mw) was determined by (4):

(4) $$\mathrm{Mw}=\frac{\left[\mathrm{CD}\right]\times N_{A}}{n}\times {\mathrm{Mw}}_{1}$$

where n is the number of particles (particles/mL determined by NTA), [CD] is the concentration of CD tori (mole/mL determined by FTIR spectroscopy), N_A_ is Avogadro’s constant, and the molecular weight of the repeating unit (CD and linker) is Mw_1_.

### 2.8. Dynamic Light Scattering

The ζ-potential determination was performed using the Zetasizer Nano S «Malvern» equipped with a 4 mW He–Ne laser (633 nm; Malvern, UK) at 25 °C. The analysis was performed 3 times for each sample using the correlation of the Correlator system K7032-09 «Malvern» (Malvern, UK). The values are provided with standard deviations.

### 2.9. The Determination of Stern–Volmer Constants and Binding Affinity (Ka) for Drug Form–HSA

The protein fluorescence quenching with small molecules is described using the Stern–Volmer Equation (5) [23], which takes into account the contribution of both statistical and dynamic quenching.

(5) $$F_{o}/=1+K_{\mathrm{SV}}[Q]=1+k_{q}\tau_{0}[Q],$$

In Equation (4), $F_{o}$ and F are the fluorescence intensities in the absence and presence of a drug form (quencher), Ksv is a Stern–Volmer constant, [Q] is the molar concentration of the quencher, $k_{q}$ and $\tau_{0}$ are the bimolecular constants of the quenching rate and the lifetime of HSA fluorescence in the absence of the quencher.

The determination of the binding constants of HSA complexes with drug forms (Ka) were determined by Equation (6) [23] at 310 K. The number of binding sites (n) was supposed to be 1.

(6) $$\log\frac{F_{o}-F}{F}={\mathrm{logK}}_{a}+\mathrm{nlog}\left[Q\right]$$

### 2.10. Circular Dichroism Spectroscopy

The circular dichroism spectra were registered on a spectrometer J-815 company «Jasco» (Tokyo, Japan) equipped with a thermostatic cell. The measurements were made within a wavelength range of 200−260 nm at 25 °C in a quartz cuvette (l = 1 mm). Spectra were scanned 5 times at 1 nm. The HSA concentration was maintained at 0.02 mM.

### 2.11. NMR Spectroscopy

A total of 10–15 mg of the CD polymer were dissolved in D_2_O, and 1H NMR spectra were recorded on a Bruker Avance 400 spectrometer (400 MHz, Reinshtetten, Germany).

### 2.12. UV-Spectroscopy

The UV spectroscopy was carried out by Ultrospec 2100 pro equipment (Amersham Biosciences, Amersharm, UK) in a wavelength range of 200–450 nm in a Hellma Analytics cell (quartz, 1 cm; Jena, Germany).

### 2.13. In Vitro Studies

We determined the minimum inhibition concentration (MIC) by the agar well diffusion method [25,26,27]. The cell’s suspension (overnight *Escherichia coli* ATCC 25,922 (grown in Luria Bertuni medium for 12 h)) was diluted to 0.5 McFarland standard. Four wells with diameters of 9 mm were cut in the agar by sterile plastic pipette tips after the distribution of bacteria over the agar surface. A total of 50 μL of the samples (sterile buffer (negative control), AM, AM–HPCD, AM–HSA, AM–HpolH (HpolC for Cef), and AM–HpolH–HSA (HpolC for Cef); C_Lev_ = 0.1, 0.2, 0.5 μg/mL and C_Cef/Mer_ = 1.0, 2.0, 5.0 μg/mL for each sample) were placed in wells. Then, the Petri dishes with samples were incubated at 37 °C. After 24 h, the diameters of the inhibition zones were analyzed. MICs were defined as the concentration of the sample at which the absence of the bacterial growth equals that of the removed agar (diameter ≈ ca. 9 mm) (7):

(7) $${\mathrm{lgC}}_{\mathrm{AM}}=a\times S_{\mathrm{inh}}+\mathrm{lgMIC}$$

where C_AM_ is AM concentration (μg/mL) and S_inh_ is the square of the inhibition zone (mm^2A^). Each sample was tested in triplicate. The data are presented with standard deviations.

## 3. Results and Discussion

**Figure 1:**
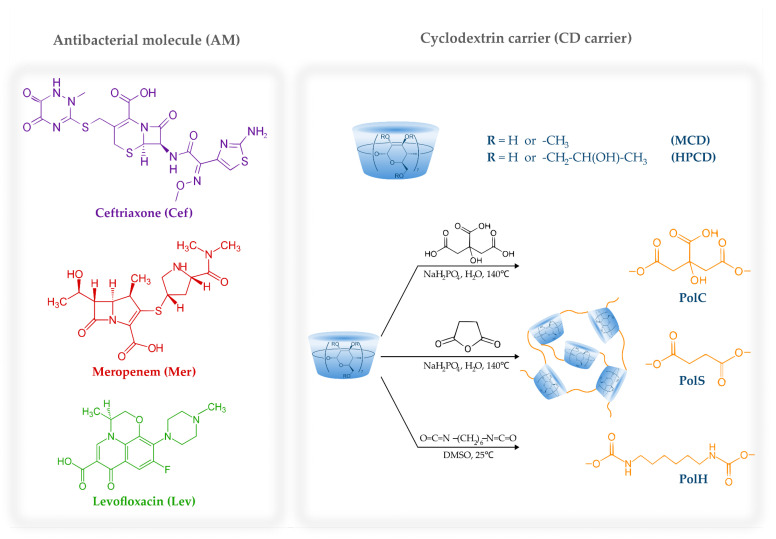
The chemical structures of ceftriaxone, meropenem, levofloxacin, HPCD, MCD and the schematic representation of CD-based polymers.

In this study, we aimed to discover the main patterns of the interaction between the antimicrobial drugs loaded onto CD carriers and HSA with a focus on the role of the carrier. We considered three drugs suitable for pulmonary infection treatment, namely, ceftriaxone (Cef), meropenem (Mer) and levofloxacin (Lev). Figure 1 presents the chemical structures of the antibacterial molecules and CDs under consideration.

Besides the commonly investigated 2-hydroxypropyl-β-cyclodextrin (HPCD) and methyl-β-cyclodextrin (MCD), we studied CD-based polyesters and polyurethanes obtained via cross-linking HPCD and MCD (Figure 1) with citric acid, succinic anhydride or 1,6-hexamethylene di-isocyanate (HMD). We have recently reported on these polymers’ physical and chemical properties [15,21], and here we summarize them in Table 1.

Briefly, CD polymers with the average Mw of 150–240 kDa formed nanoparticles (120–200 nm in diameter) in aqueous media. Their ζ-potentials depended on the linker type: in the case of citric acid, we supposed a negative charge due to unreacted –COOH groups on the particle’s surface, whereas for the bifunctional linker succinic anhydride, a neutral charge; cross-linking with di-isocyanate led to the hydrolysis of unreacted terminal –N=C=O groups to primary amines that determined the polymers’ positive ζ-potential. The structures of the polymers were confirmed by FTIR and 1H NMR. The degree of CD cross-linking was established by the normalization of the CD polymers’ NMR spectra to the H1 peak of CD: ca. 3–4 linkers per CD torus for all the samples.

Thus, the carriers under consideration possessed suitable physical–chemical characteristics, and first we aimed to investigate the AM–CD complex formation.

### 3.1. AM Interaction with CD and CD Polymers

The guest–host complexes of CD derivatives with Cef, Mer and Lev are well-studied in the literature [28,29,30,31]. For AM–CD complexes, the average binding constant (Ka) values are near 10^2^–10^3^ M^–1^, whereas for drug–CD polymers they might reach 10^5^ M^–1^ [15]. Since we have recently studied the molecular aspects of HPCD complexes with Lev [32], it was interesting to investigate how the polymeric network might influence the binding affinity.

**Figure 2:**
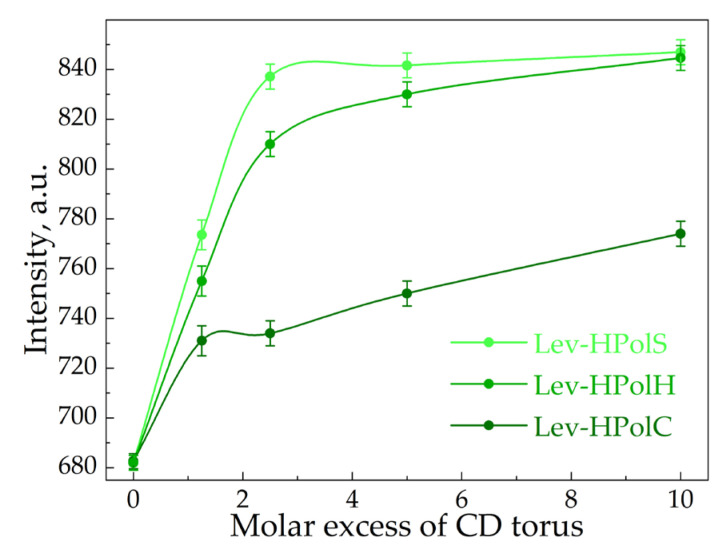
The dependence of the intensity of levofloxacin’s fluorescence emission on the CD torus molar excess in CD polymers, λ_ex_ = 458 nm, C_0_(Lev) = 4.8 μM.

For the determination of Ka values, we used fluorescence spectroscopy. At λ_ex_ = 345 nm, Lev demonstrated a peak with a maximum at 458 nm corresponding to the π* → π transitions. In the presence of all the CD polymers, the intensity of the peak rose with the increase in the carrier’s molar excess (Figure 2), which is typical for drug–CD complexes [33]. Interestingly, we did not observe the pronounced saturation for Lev complexes with HpolC and HpolS, whereas in the case of Lev–HpolH the plateau appeared at a CD torus molar excess of 0.5. Thus, probably only some of the HpolH binding sites were available for interaction. Apparently, the positive ζ-potential of the particle repelled Lev’s positive charge in the heterocycle. In the case of HpolC and HpolS, no saturation indicated that not only were the CD tori involved in binding, but also that there were some additional binding centers, possibly unreacted –COOH groups of the polymer.

Table 2 and Figure S1 show the Ka values calculated via Hill’s analysis. Indeed, comparing Ka_Lev–CD_ with Ka_Lev–CDpolymers_, one observes the significant impact of the polymeric network on the complexes’ stability. HpolS and HpolH exhibited higher Ka values than HpolC. We supposed that methylene groups on the linker were involved in the interaction with Lev molecules. Hence, the hydrophobic fragments and a partial small charge on the surface are factors that enhanced the binding efficiency.

Thus, CD-based polymers surpassed CDs in molar mass, size and affinity for drug molecules, and we expected that CD polymers might affect AM–HSA binding more significantly than CDs. Hence, we begin our discussion with the AM and AM–CD interactions with HSA, and further consider the AM–CD polymer–HSA complexes separately.

### 3.2. AM and AM–CD Interactions with HSA

#### 3.2.1. HSA Fluorescence Quenching Study

**Figure 3:**
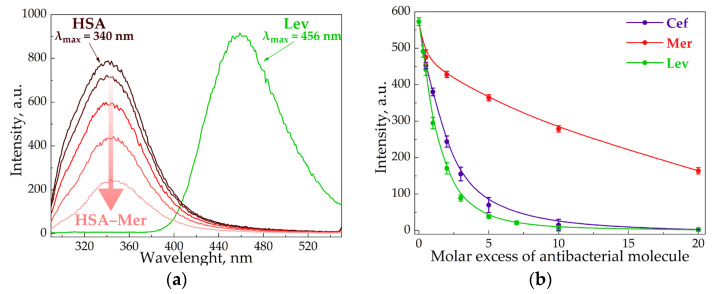
Emission spectra of Lev, HSA and HSA–Mer at λ_ex_ = 280 nm, C_HSA_ = 0.02 mM, C_Mer_ = 0.01–0.1 mM, C_Lev_ = 0.01 mM, pH 7.4, and 37 °C (**a**). The intensity of HSA peak via the increase in AM concentration at λ_ex_ = 280 nm, C_HSA_ = 0.02 mM, pH 7.4, and 37 °C (**b**).

In order to disclose the role of the CD–carrier in the interaction of AM with HSA, we used fluorescence spectroscopy, which is widely applied to complex samples, including biological samples [34,35]. Tryptophan (Trp), tyrosine and phenylalanine amino acid residues induce HSA fluorescence [36,37]. At λ_ex_ = 280 nm, HSA exhibited an emission band at 340 nm (Figure 3a), while Cef and Mer had no emission spectra at such conditions. Thus, the AM and HSA spectra did not overlap, enabling us to observe the albumin’s state during the interaction with the drug molecules.

All the studied AMs significantly decreased the HSA fluorescence intensity (Figure 3b), which indicates that albumin complexation with the AM caused the change in Trp’s microenvironment. Moreover, the quenching strengthened as the AM concentration increased. Indeed, several research groups reported the similar results for other drug–HSA systems [38,39,40]. For the quantitative analysis of HSA fluorescence quenching, we calculated the Stern–Volmer constant Ksv (Table 3, Figure S2), and for the binding efficiency, the Ka (Table 4, Figure S3). Ksv allows the evaluation of fluorophore quenching by small molecules, taking into account both statistical and dynamic quenching. For small molar excesses, only statistical quenching is observed [29].

The Ksv and Ka values for AM–HSA were in good agreement with the literature data [23,41,42]. It is worth noting the significance of albumin’s quenching lowering for Lev > Cef >> Mer. The most hydrophobic Lev molecule apparently bound to HSA most effectively via hydrophobic interactions with Trp, which contributed greatly to HSA fluorescence. Indeed, for Lev–HSA, the position of the albumin’s emission maximum shifted to the long-wavelength region by about 10 nm. This may additionally indicate that the Trp residue appears in a less hydrophobic environment [43]. Similar changes were not observed in the case of Mer and Cef, which indicated less effective complexation with HSA.

Let us discuss three-component systems (AM–CD carrier–HSA). First, we confirmed that CDs and their polymers (0.01–1 mM) did not influence the HSA emission spectra, indicating the absence of pronounced changes in the protein structure via binding [44]. Nevertheless, many authors state that CDs weaken the quenching and binding affinity between the drug and HSA, as well as bovine serum albumin (BSA), without changing either the binding site or binding force between the drug and albumin. The authors suppose that this effect is associated with the retention of the drug in the guest–host complex, i.e., the protein and CD compete to bind free drug molecules [45,46,47,48]. Thus, we expected similar trends for the AMs under consideration. Indeed, in the case of the Lev–CD–HSA systems, the Ksv and Ka values were slightly lower compared to the two-component systems (Table 3 and Table 4).

In contrast, for hydrophilic drug molecules, the opposite trends were observed. According to the Ka values, Cef’s binding affinity for HSA was 10^3^ times higher than for CDs. This means that the protein won the competition for Cef’s binding. The increase in Ka (for Cef–CD–HSA) might be due to CD’s interactions with the albumin, which induce changes in the protein structure that are favorable for more efficient Cef binding.

What is more interesting, Ka_Mer–CD_ and Ka_Mer–HSA_ are close; however, the three-component systems demonstrated a dramatic increase in the drug’s binding affinity (by 10^3^ times). For the discussion of this unexpected discovery, let us consider the drug–HSA complex in depth.

The main drug binding sites on albumin are well-studied; the cavities with similar chemical properties include site I (subdomain IIA) and site II (subdomain IIIA) [49,50]. Lev binds at both sites with equal affinity [51], whereas more polar molecules demonstrate higher selectivity; Cef prefers site I [52] and Mer favors site II [42]. On the contrary, CD’s binding site in HSA is still under consideration. For β-CD, Ghosh S. et al. claim subdomain IIB [44], whereas more recent studies state subdomain IB [36]. Moreover, the authors reveal that CD’s size and substituent alters the binding site; for instance, HPCD and dimethyl–CD are located in IIIA. Nevertheless, both studies report that CDs interact with the HSA amino acids located on the albumin surface, i.e., they do not integrate into protein moiety. The CD–albumin interaction is rather weak (for β-CD–HSA Ka is 185 M^–1^).

These observations are essential for understanding our data, especially in the case of Mer–CD–HSA. Briefly, Ka_Mer–CD_ ≈ Ka_Mer–HSA_ ≈ Ka_CD–HSA_, and both Mer and CDs have the same binding site on HSA (subdomain IIIA). Thus, we supposed that the significant increase in Mer’s binding to protein in the three-component system might be explained by the fact that after Mer bound to HSA, CDs covered the bound drug from above as a “lid”.

#### 3.2.2. The Analysis of HSA’s Secondary Structure

Several studies report that the formation of the drug–HSA complex leads to changes in albumin’s secondary structure [53,54,55]. We conducted similar investigations for our two-component and three-component systems using commonly applied FTIR and circular dichroism spectroscopy.

**Figure 4:**
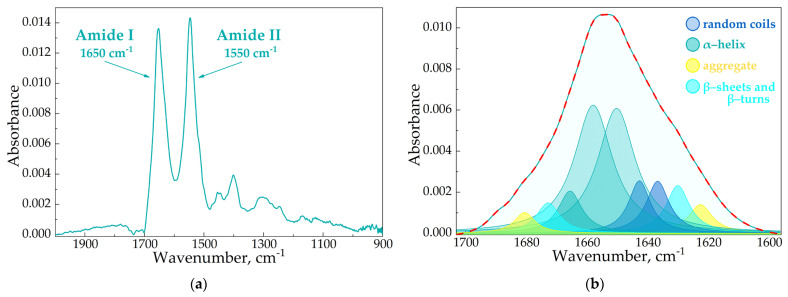
The FTIR spectra of HSA, pH 7.4 (**a**). The deconvolution of HSA FTIR spectra, C_HSA_ = 0.06 mM, simulated spectra marked in red, pH 7.4 (**b**).

In the FTIR spectra of HSA, we observed two specific bands corresponding to the oscillations of peptide bonds. The amide I (1600–1700 cm^–1^) band represents the oscillation of ν(C=O)~80% and ν(C-N)~15%, while the amide II (1500–1600 cm^–1^) bands correspond to δ(N-H)~60%, ν(C-N)~20% and (C-C)~10% [56,57,58]. Both bands were sensitive to the changes in the protein secondary structure, and the amide I analysis provided important insights into the changes in the protein’s secondary structure. The deconvolution of this band uncovered the contents of α-helices, β-structures (β-turns and β-sheets) and random coils (Figure 4).

In the native protein, ~65% α-helices were presented (Table 5), which correlated with the literature data as well as the X-ray structure [41,59]. CDs and CD polymers did not induce pronounced changes in HSA’s secondary structure. In contrast, the AM caused a decrease in α-helices (10–20%), mainly due to the increase in β-structures and random coils, which is typical for other drug–HSA systems [41,60,61]. Interestingly, changes in the HSA structure were almost equal for Lev and Cef; however, in the case of Mer the effect strengthened by 2 times, although Ka_Mer–HSA_ was the lowest. Thus, the data from the fluorescence studies and the values Ka were in the clear agreement with the FTIR analysis.

Considering three-component systems (AM–CD–HSA), we observed similar changes in protein structure. Nevertheless, the magnitude of the effect depended on the AM. For Lev–CD, the altering of HSA’s structure was compared to the two-component system. Thus, CD slightly altered Lev–HSA binding (binding affinity and HSA’s structural changes). Apparently, CD prevented the binding of Lev’s albumin binding site via competition with HSA.

The data in the case of Cef were more interesting: AM–CD induced a greater decrease in α-helix content (to 40%) and an increase in β-structures by ~37%. These changes might be the reason for the Ka increase for the three-component system (Table 4). For Mer, the content of albumin’s secondary structures was comparable for Mer–HSA and Mer–CD–HSA, thus the higher Ka_Mer–CD–HSA_ value might be explained by the fact that CDs blocked the drug dissociation of the protein, as we discussed above.

**Figure 5:**
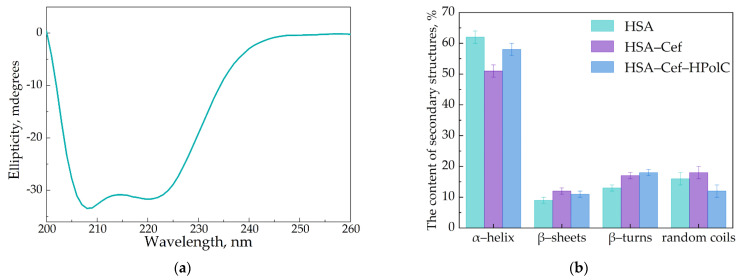
Circular dichroism spectrum of HSA, C = 0.05 mM, pH 7.4 (**a**). The content of secondary structures in HSA, C_HSA_ = C_Cef_ = C_CDtori_ = 0.02 mM, pH 7.4 (**b**).

Circular dichroism spectroscopy was used to confirm the structural changes in HSA. The albumin demonstrated two negative peaks at 208 and 222 nm [62]. The data obtained by the measurements were in good agreement with the FTIR results (Figure 5).

### 3.3. AM–CD Polymers Interactions with HSA

CD polymers might affect AM–HSA interactions more significantly, since they possess higher mass, size and demonstrate a higher affinity for AM (Table 2). A few papers discuss the binding affinity and structural changes in albumin via protein–nanoparticle complex formation [63,64]. We found that CD polymers interacted with HSA in the same manner as CDs: no pronounced changes in HSA’s fluorescence spectra or protein secondary structures, which means that CD polymers had a mild effect on albumin. This result is promising for their biomedical application, because some nanoparticles—for example, dendrimers—induce partial HSA unfolding [65].

For all the AM–CD polymer–HSA complexes, we observed a dramatic increase in drug–albumin binding affinity (Table 4, Figure S3): compared to AM–HSA, the Ka values increased by 1–3 orders of magnitude. Interestingly, for Cef the most pronounced effect was demonstrated by HpolC, while Lev–CD polymers linked with di-isocyanate. Since Lev is less hydrophilic (Figure 1), the preference for the polymer with hydrophobic pores is reasonable, as well as the opposite trend for the more hydrophilic Cef molecule. In the case of Mer, no predominance was observed that might be explained by Mer’s limited aqueous solubility. Nevertheless, all CD polymers demonstrated a greater influence of AM–HSA than CDs.

The significant increase in Ka might be explained by the high affinity of AM for CD polymers, which led to the strong drug retention in the CD polymer–HSA complex. Additionally, we have recently demonstrated that CD carriers slow down moxifloxacin and meropenem release from AM–CD carrier complexes in 24 h [15,21]. Thus, in the case of AM–CD polymer–HSA complexes, only a low content of free drug molecules was present in the solution. On the one hand, this result seems to be undesirable. However, several studies report that polymeric nanoparticles are beneficial in achieving prolonged drug release. Since nanoparticles interact with albumin and form a shell–core structure, the released drug molecule initially binds to HSA, which might significantly lower the content of free AM in the blood and consequently prolong the therapeutic effect [66,67].

Interestingly, the content of HSA’s secondary structures was slightly lower than that of HSA’s free form. The absence of severe albumin conformation changes confirmed that the CD polymer won the competition for drug binding.

The high Ka values for the AM–CD polymer–HSA complexes might lead to the lack of free drug molecules, so the systems might possess low antibacterial activity. To uncover the in vitro properties of the three-component systems, we conducted microbiological studies.

### 3.4. Antibacterial Activity of AM Drug Forms in the Presence of HSA

**Figure 6:**
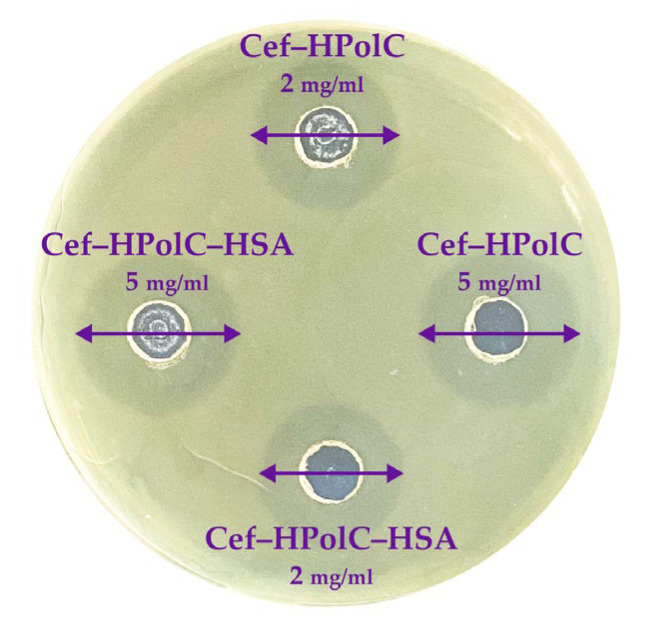
The results for antibacterial activity of CD polymers (37 °C) agar well diffusion method, *E. coli* ATCC 25922.

We investigated how HSA affects the AM and AM–CD carrier’s antibacterial activity using the Gram-negative bacteria *Escherichia coli* ATCC 25922 by the fast and robust agar well diffusion method (Figure 6) [68]. First, we determined the minimum inhibition concentration (MIC) for AM (Table 6). The MIC_AM_ values were in good agreement with the literature data [69,70,71]. As expected, CDs and all the CD polymers did not affect bacterial growth, which supported our previous data [15]. Furthermore, the AM–CD carriers acted comparably to the free AM, which indicates that all the non-covalent complexes released all drug molecules in 24 h.

Albumin proved to be non-toxic and biocompatible [72]; thus, let us consider the three-component systems. We found that HSA did not alter the MIC values for either type of CD carrier. Interestingly, even the samples with a Ka up to 108 M^–1^ demonstrated the same in vitro activity as the free AM forms. As we discussed above, the drug’s encapsulation in the CD carriers slowed down the drug release rate, and 100% of the drug was released in 24 h. Thus, we supposed that due to diffusion, comparable inhibition zones appeared. Nevertheless, all the AM–CD carrier–HSA complexes demonstrated satisfying antibacterial activity, so HSA did not decrease the potential microbiological properties of the drug form.

## 4. Conclusions

Bacterial infection diseases and the COVID-19 pandemic still threaten the public and medical communities worldwide. Antibiotics and antibacterial agents are commonly used for patients in hospitals; however, the emerging antimicrobial resistance and undesirable side effects pose a serious challenge for researchers to design more efficient drug forms with prolonged releases.

In this work, we investigated how the prospective drug carriers cyclodextrins (CDs) and CD-based polymers might influence drug interactions with human serum albumin (HSA). The formation of drug–CD non-covalent complexes slightly altered the drug’s affinity for HSA among albumin’s conformations. The most pronounced increase in binding affinity was observed for meropenem. We supposed this effect might be explained by the fact that Mer demonstrated comparable binding affinity for both CD and HSA, and both Mer and CDs have subdomain IIIA binding site on HSA. Thus, after Mer’s binding to HSA CDs blockers drug’s dissociation from the albumin.

For the first time, drug–CD polymer–HSA systems were studied. We found that CD polymers minor protein conformation changes via the AM binding compared to CDs. Moreover, the significant increase in binding affinity (up to 10^2^ times) was observed for all studied drugs. We suppose this effect might lead to the pronounced decrease in the drug release rate from drug–CD polymer–HSA complexes in blood plasma after intravenous administration, enhance the drug’s lifetime in vivo, and consequently prolong the therapeutic effect. Furthermore, these data might also be interesting for the development of other drug forms that contact the blood; for instance, wound healing and implant coating CD-based polymers. The drug–CD carrier–HSA complexes demonstrated comparable antibacterial activity in vitro to the drug’s free forms. Even the high Ka values did not decrease the drug’s microbiological properties in 24 h.

Besides HSA, other blood plasma components might also affect drug–carrier properties, so further research must be performed for more detailed formulation behaviors in blood plasma and in vivo. Nevertheless, according to this study, we suppose that CD polymers are more promising drug carriers than CDs for the design of new drug forms with prolonged releases.

## Figures and Tables

**Table 1:** Physical and chemical characteristics of CD-based polymers [15,21].

CD Type	Linker	Abbreviation ^1^	Dh, nm	ζ-Potential, mV
HPCD	Citric acid	HpolC	120.1 ± 3.2	– ^2^
	Succinic anhydride	HpolS	199.2 ± 18.2	5.6 ± 2.1
	HMD	HpolH	142.5 ± 13.5	14.7 ± 0.4
MCD	HMD	MpolH	170.3 ± 14.2	30.8 ± 1.1

^1^ The abbreviation consists of “CD type–polymer–linker type”. ^2^ ζ-potential of HpolC was not determined since a decarboxylation reaction may occur under the experimental conditions.

**Table 2:** Ka values (M^–1^) for Lev–CD carriers, 37 °C.

Lev–HPCD	Lev–HpolC	Lev–HpolS	Lev–HpolH
1.0 (±0.3) × 10^3 1^	1.2 (±0.3) × 10^3^	2.2 (±0.4) × 10^5^	1.1 (±0.2) × 10^5^

^1^ [32].

**Table 3:** Ksv values (M^–1^) for AM–HSA and AM–CD carrier–HSA (0.02 M sodium phosphate buffer solution, pH 7.4, 37 °C).

	Ceftriaxone	Meropenem	Levofloxacin
–	3.8 (±0.1) × 10^4^	0.57 (±0.01) × 10^4^	3.4 (±0.2) × 10^4^
HPCD	3.0 (±0.3) × 10^4^	0.85 (±0.02) × 10^4^	3.3 (±0.1) × 10^4^
MCD	4.9 (±0.2) × 10^4^	0.93 (±0.02) × 10^4^	3.1 (±0.1) × 10^4^
HpolC	1.9 (±0.2) × 10^5^	2.1 (±0.2) × 10^4^	7.7 (±0.4) × 10^4^
HpolS	2.7 (±0.2) × 10^5^	2.4 (±0.2) × 10^4^	1.1 (±0.3) × 10^5^
HpolH	2.0 (±0.2) × 10^5^	1.3 (±0.1) × 10^4^	9.8 (±0.4) × 10^4^
MpolH	1.9 (±0.4) × 10^5^	1.8 (±0.1) × 10^4^	1.7 (±0.2) × 10^5^

**Table 4:** Ka values (M^–1^) for AM–HSA and AM–CD carrier–HSA (0.02 M sodium phosphate buffer solution, pH 7.4), 37 °C.

	Ceftriaxone	Meropenem	Levofloxacin
–	1.7 (±0.1) × 10^5^	1.1 (±0.1) × 10^2^	9.9 (±0.3) × 10^5^
HPCD	2.0 (±0.2) × 10^5^	3.6 (±0.2) × 10^4^	3.5 (±0.1) × 10^5^
MCD	3.3 (±0.3) × 10^5^	9.7 (±0.1) × 10^3^	8.9 (±0.2) × 10^4^
HpolC	3.8 (±0.4) × 10^8^	8.4 (±0.4) × 10^5^	9.4 (±0.4) × 10^5^
HpolS	2.9 (±0.3) × 10^7^	4.9 (±0.3) × 10^5^	3.9 (±0.2) × 10^6^
HpolH	9.1 (±0.2) × 10^6^	9.3 (±0.1) × 10^5^	7.0 (±0.3) × 10^6^
MpolH	4.6 (±0.3) × 10^6^	3.5 (±0.1) × 10^5^	6.9 (±0.2) × 10^6^

**Table 5:** The content of secondary structures in HSA, C_HSA_ = C_AM_ = C_CDtori_ = 0.06 mM, pH 7.4, SD (n = 3).

	α-Helix	β-Sheets	β-Turns	Random Coils
HSA	64 ± 3	8 ± 0.5	12 ± 1	16 ± 1
Cef–HSA	52 ± 2	13 ± 1	16 ± 1	18 ± 2
Cef–MCD–HSA	41 ± 2	14 ± 1	22 ± 1	23 ± 2
Cef–HpolC–HSA	60 ± 2	10 ± 1	11 ± 1	18 ± 2
Mer–HSA	39 ± 2	15 ± 1	21 ± 2	26 ± 2
Mer–MCD–HSA	40 ± 2	16 ± 1	22 ± 2	21 ± 2
Mer–HpolH–HSA	55 ± 2	13 ± 1	17 ± 2	24 ± 2
Lev–HSA	55 ± 2	9 ± 1	15 ± 1	22 ± 2
Lev–HPCD–HSA	57 ± 2	14 ± 1	14 ± 1	23 ± 2
Lev–MCD–HSA	58 ± 2	14 ± 1	9 ± 1	20 ± 2
Lev–HpolH–HSA	60 ± 2	13 ± 1	10 ± 1	17 ± 2

**Table 6:** MIC (µg/mL), pH 7.4 (PBS sterile buffer), agar well diffusion method, 37 °C.

Sample Type	CD Carrier	Cef	Mer	Lev
AM	*–*	0.17 ± 0.3	0.12 ± 0.1	0.08 ± 0.02
AM–CD carrier	HPCD	0.17 ± 0.2	0.13 ± 0.2	0.06 ± 0.02
	HpolC	0.16 ± 0.2	0.11 ± 0.2	0.07 ± 0.02
AM–HSA	–	0.18 ± 0.3	0.14 ± 0.2	0.09 ± 0.02
AM–CD carrier–HSA	HPCD	0.18 ± 0.3	0.14 ± 0.2	0.07 ± 0.01
	HpolC	0.18 ± 0.2	–	–
	HpolH	–	0.14 ± 0.2	0.08 ± 0.02

## Data Availability

All data generated or analyzed during this study are included in this published article and its Supplementary Information.

## Supplementary Materials

The following supporting information can be downloaded at: https://www.mdpi.com/article/10.3390/pharmaceutics15061598/s1, Figure S1: The linearization of the changes in the Lev’s emission spectra intensities via complex formation with CD carriers in Hill’s coordinates; Figure S2: The determination of Ksv values (M–1) for AM–HSA and AM–CDcarrier–HSA by the Stern-Volmer equation, 0.02 M sodium-phosphate buffer solution, pH 7.4, 37 °C; Figure S3: The determination of Ka values (M–1) for AM–HSA and AM–CDcarrier–HSA, 0.02 M sodium-phosphate buffer solution, pH 7.4, 37 °C.
