# Supplementary material for: Cyclodextrins and Their Polymers Affect Human Serum Albumin’s Interaction with Drugs Used in the Treatment of Pulmonary Infections
Source: Pharmaceutics. 2023 May 25;15(6):1598. doi: 10.3390/pharmaceutics15061598 (PMC10301439; doi:10.3390/pharmaceutics15061598)
Supplement: Supplementary file 1 [file pharmaceutics-15-01598-s001.zip › pharmaceutics-2366452-supplementary.pdf]

pharmaceutics

Article

Cyclodextrins and Their Polymers Affect Human Serum Albumin's Interaction with Drugs Used in the Treatment of Pulmonary Infections

Anna A. Skuredina, Linara R. Yakupova, Tatiana Yu. Kopnova, Irina M. Le-Deygen, Natalya G. Belogurova and Elena V. Kudryashova *

Supplementary

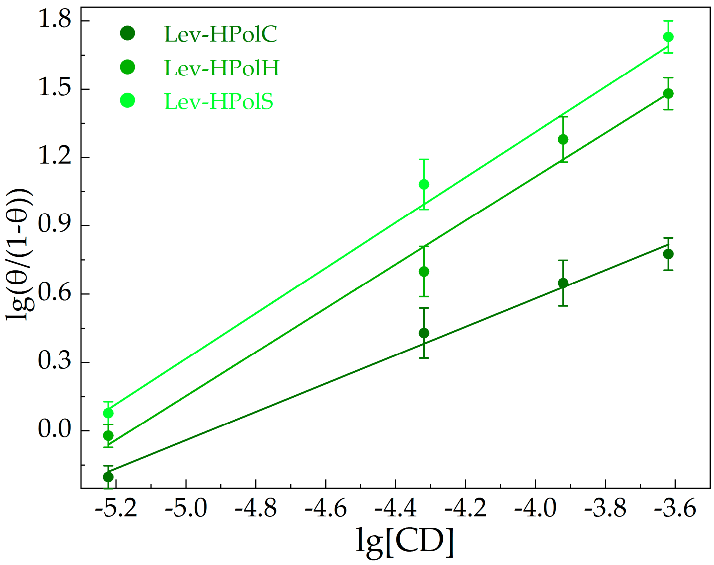

Figure S1 The linearization of the changes in the Lev's emission spectra intensities via complex formation with CD carriers in Hill's coordinates.

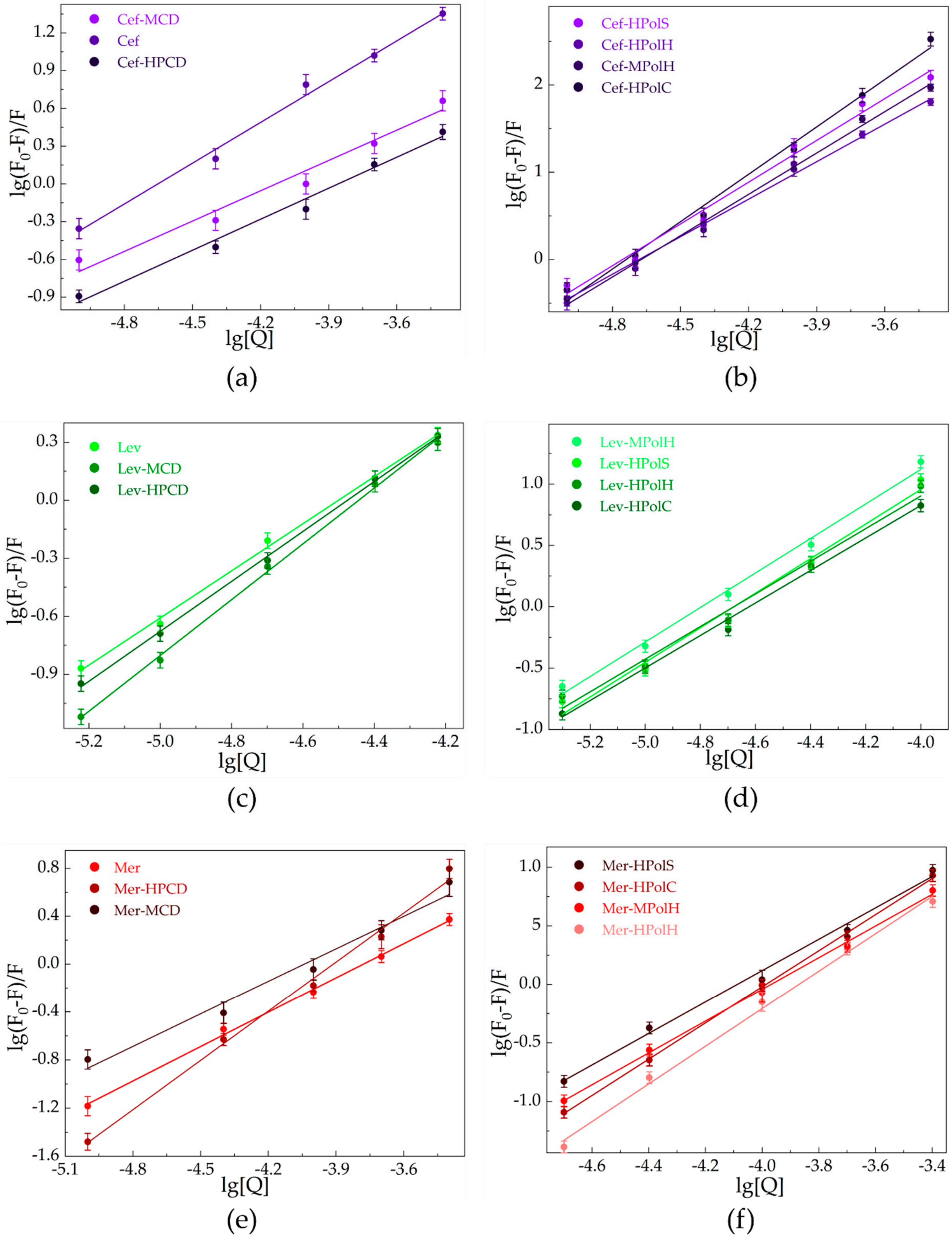

Figure S2 The determination of Ksv values (M^{-1}) for AM–HSA and AM–CDcarrier–HSA by the Stern-Volmer equation, 0.02 M sodium-phosphate buffer solution, pH 7.4, 37 °C.

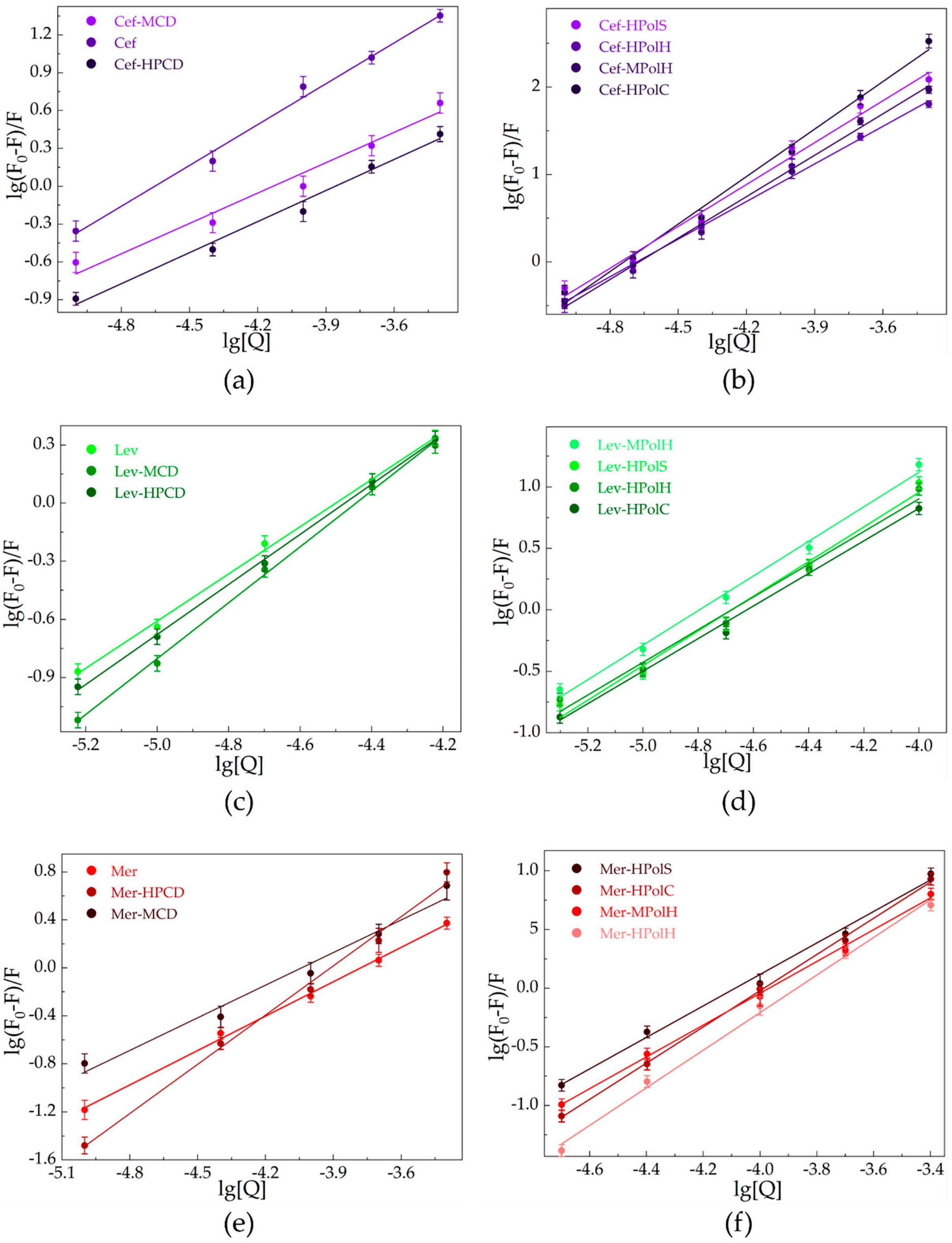

Figure S3 The determination of Ka values (M^{-1}) for AM–HSA and AM–CDcarrier–HSA, 0.02 M sodium-phosphate buffer solution, pH 7.4, 37 °C.
